# Two Complementary Personal Medication Management Applications Developed on a Common Platform: Case Report

**DOI:** 10.2196/jmir.1815

**Published:** 2011-07-12

**Authors:** Stephen E Ross, Kevin B Johnson, Katie A Siek, Jeffry S Gordon, Danish U Khan, Leah M Haverhals

**Affiliations:** ^5^Colorado REAP (Research Award Enhancement Program) to Improve Care CoordinationVeterans Affairs Medical CenterDenver, COUnited States; ^4^School of NursingVanderbilt UniversityNashville, TNUnited States; ^3^Wellness Innovation and Interaction LabDepartment of Computer ScienceUniversity of Colorado BoulderBoulder, COUnited States; ^2^Department of Biomedical InformaticsVanderbilt UniversityNashville, TNUnited States; ^1^Division of General Internal MedicineSchool of MedicineUniversity of Colorado Anschutz Medical CampusAurora, COUnited States

**Keywords:** Health records, personal, drug information services, medical informatics

## Abstract

**Background:**

Adverse drug events are a major safety issue in ambulatory care. Improving medication self-management could reduce these adverse events. Researchers have developed medication applications for tethered personal health records (PHRs), but little has been reported about medication applications for interoperable PHRs.

**Objective:**

Our objective was to develop two complementary personal health applications on a common PHR platform: one to assist children with complex health needs (MyMediHealth), and one to assist older adults in care transitions (Colorado Care Tablet).

**Methods:**

The applications were developed using a user-centered design approach. The two applications shared a common PHR platform based on a service-oriented architecture. MyMediHealth employed Web and mobile phone user interfaces. Colorado Care Tablet employed a Web interface customized for a tablet PC.

**Results:**

We created complementary medication management applications tailored to the needs of distinctly different user groups using common components. Challenges were addressed in multiple areas, including how to encode medication identities, how to incorporate knowledge bases for medication images and consumer health information, how to include supplementary dosing information, how to simplify user interfaces for older adults, and how to support mobile devices for children.

**Conclusions:**

These prototypes demonstrate the utility of abstracting PHR data and services (the PHR platform) from applications that can be tailored to meet the needs of diverse patients. Based on the challenges we faced, we provide recommendations on the structure of publicly available knowledge resources and the use of mobile messaging systems for PHR applications.

## Introduction

Medication management accounts for the majority of medical errors in ambulatory care [1,2]. Errors in home administration account for many of these errors [3-5], particularly after care transitions, such as being discharged home from the hospital [6,7]. Some errors occur because patients commonly have difficulty maintaining an accurate, current list of increasingly complex medication regimens [8,9]. Lay persons find generic and proprietary medication names to be inscrutable and redundant. Patients also lack critical information about the medicines they take. In busy practices, clinicians only inconsistently review medication regimens and warn about the potential side effects of new medications [10,11]. Even with assistance from pharmacists and resources such as medication information sheets, many patients remain uncertain about key medication questions [12].

The Institute of Medicine recommends patient-centered approaches to address these deficiencies: fostering a strong consumer–provider partnership in medication management, enhancing communication, and developing tools for “patient (or surrogate) self-management support” [2]. Groups such as the Markle Foundation [13] and the Commission for Systemic Interoperability [14] note the role personal health records (PHRs) can play in improving medication management. Paper PHRs are familiar in pediatric practice [15,16] and improve medication management in adult care transitions [17,18]. Electronic PHRs have also shown promise as aids to medication management [2,19]. These include *standalone* PHRs such as MyMedicationList, which links patient-entered medication data to consumer health information [20], and *tethered* PHRs such as the Patient Gateway medications module, which allows patients to review, track, and communicate with physicians about the medication list derived from a leading institution’s electronic medical record [19].

*Interoperable* PHRs promise to empower patients even more. For medication management, an interoperable platform for PHR data could improve coordination of care by consolidating multiple sources of prescribing data (from the electronic medical records of multiple independent practices) and fulfillment or dispensing data (from pharmacy records and claims), allowing patients to share these data at their discretion [14,21,22]. An application layer could enrich these data with consumer health information [23], tools that identify drug interactions and duplications, and scheduling applications. Mobile applications could support reminders to take medications and facilitate communication among patients and caregivers. However, with these advantages also come the daunting challenges of designing devices and user interfaces that are reliable, are easy to use, and present complex information in ways that consumers find straightforward and helpful.

As part of Project HealthDesign [24] we explored these opportunities and challenges. Project HealthDesign was launched in December 2006 (before the availability of commercial PHR platforms such as Indivo, Microsoft HealthVault, and Google Health) to “demonstrate the power and potential” of interoperable PHRs. Nine teams participated in the project, each representing a different target user and use case. Our two teams had a complementary focus on medication management for patients with chronic diseases. The Vanderbilt University team developed MyMediHealth (MMH) for children with complex illnesses [25]. The University of Colorado team developed the Colorado Care Tablet (CCT) for older adults prone to care transitions [26]. While each application tailored its user interface for its target population, each used a common interoperable PHR platform [27]. Here we report how the two applications shared common services and how we addressed key informatics and user interface challenges related to ambulatory medication self-management.

## Methods

### Iterative Development Process

For all Project HealthDesign grantees, the primary objective was to create a personal health application that would be compelling for the targeted user group. The target users of MMH were children with complex diseases such as cystic fibrosis and their caregivers (parents or guardians). The target users of CCT were older patients with multimorbidity (2 or more chronic diseases such as diabetes, hypertension, or heart failure) taking multiple medications. These adults are prone to fragmentation of care through minor care transitions (seeing doctors with separate medical records systems) and major care transitions (transitions to and from the hospital).

During a 6-month design phase, project teams developed functional requirements based on a series of individual in situ interviews (eg, home, school, day care) and facilitated group discussions with target users. While a detailed description of the data collected and the analysis methods used is beyond the scope of this report, a brief description of the interviews and settings used is provided in Table 1. During the 12-month prototype phase, both groups employed iterative, user-centered design techniques to evaluate prototypes and provide the target population with a continuous voice in the design cycle. The methods employed during all phases of the project were approved by the Institutional Review Boards at the University of Colorado and Vanderbilt University.

**Table 1:** Users and settings studied in user-centered design process

Vanderbilt: MyMediHealth		Colorado: Colorado Care Tablet		
Design phase		Design phase		
	3 group sessions with parents of children with cystic fibrosis, school officials, before/after school care staff, and school nurses		12 individual interviews in home setting, 1 in hospital setting, with 15 primary users aged 73-90 years (mean 82) and 2 family caregivers aged 48 and 57	
	3 day care site visits		4 group interviews with 27 primary users over age 65 years	
	4 school site visits			
Prototype phase		Prototype phase		
	2 group sessions with parents, school officials, before/after school care staff, and school nurses		Review of storyboard	
	1-month pilot of paging system with 20 children who had cystic fibrosis			6 individual sessions with 7 participants, 5 older adults from the target user group aged 70–85 years (mean 75) and 2 caregivers aged 75 and 82 years
	Storyboard review by 200 families of children with daily chronic medication needs			2 group sessions with 9 older adults from the target user group aged 80–88 years (mean 83) and 3 caregivers aged 48–59 years (mean 53)
			6 rapid iterative testing and evaluation sessions [28,29] with a total of 22 primary users aged 61–86 years (mean 76) and 9 caregivers aged 41–61 years (mean 53)	

### Architecture: Shared Components

**Figure 1:**
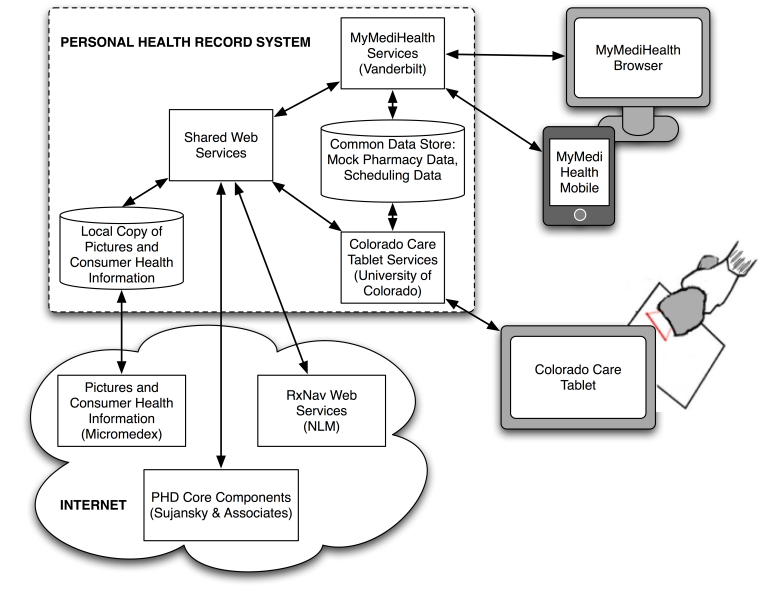
Architecture of two personal medication management applications

Early in the development process, it was clear that both applications would need a common data store and shared functions to (1) normalize medication identities (ie, translating between National Drug Codes [NDCs], RxNorm concept unique identifiers [RXCUIs], and proprietary identifications to identify duplicate medications and ingredients in medication lists), (2) link medications to consumer health information, and (3) link medications to images wherever possible. We employed a shared knowledge approach to take advantage of efficiencies in development and to provide users the ability to switch between the two applications (eg, to use CCT to build a medication list and MMH to schedule and prompt medication use). The client-server architecture, shown in Figure 1, used thin clients (off-loading the computing software to the PHR system) to make for robust, flexible, and scalable prototypes.

The *PHR platform* (PHD Core Components) used a service-oriented architecture to authenticate users and to store and retrieve various data types [27]. Of note, these prototypes did not receive, transmit, or store medication data for actual patients. MMH and CCT used simulations of electronic health record-based prescribing data and (in the case of CCT) dispensing data (ie, data available from pharmacy and claims) to test user interface scenarios. MMH also used its own local storage for timing of alerts and recording medication administration events.

In addition to the common platform, CCT and MMH used RxNav Web services [30,31] from the National Library of Medicine for normalization of medications. A commercial medication knowledge base (Micromedex; Thomson Reuters, Greenwood Village, CO, USA) was used to supply images of medications and consumer health information. Although we endeavored to use open-source tools wherever possible, no publicly available content was available for these items at the time of development.

### Medication Identity: Representation and Linkage to Knowledge Bases

The PHR platform allowed for flexibility in representations. Each medication record could be represented by a coding scheme (eg, “RXCUI”) and a code (eg, “20610”). Text entries such as generic names, trade names, and free text entries (eg, “blue pill”) were also allowed, since the target populations commonly thought of their medications by color and context. However, only 1 code could be associated with each record.

Ultimately, whenever possible we stored medication identities as NDCs. The NDC is widely used for representing medications in electronic prescribing and fulfillment data (eg, data from Surescripts medication history) and allows images to be associated with medication identities. We were able to use RxNav Web services to normalize NDC representations whenever a more abstract concept (such as the medication ingredient) was needed, but this required additional processing for normalization. In the future, storing multiple representations in each medication record (eg, storing various RXCUIs in addition to the NDC, as is possible with the ASTM Continuity of Care Record and HL7 Continuity of Care Document schema) in advance would reduce processing cycles when the application is run.

**Figure 2:**
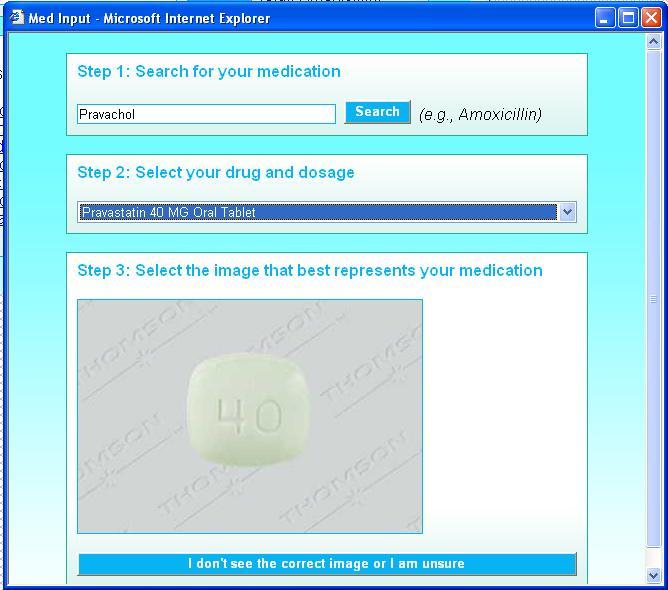
Medication selection user interface for both applications

Recognizing that some medications would need to be entered manually by users, we developed systems to assist capture of codified data, rather than simple free text. CCT and MMH employed parallel functionality for this purpose, as shown in Figure 2. When users typed in part or all of a medication name, the application displayed a list of candidate medications. When corresponding images existed, they were presented to the patient. When the user confirmed the image of the medication to be entered, the associated NDC was stored as the medication identity. If the name of the medication matched, but none of the images matched, then the application could not derive an NDC, and the RXCUI associated with the semantic clinical drug name was stored. This functionality was supported using RxNav services and the Micromedex drug image database:

- The text string entered by the user was processed by the RxNav spell check function. If the name was not recognized, alternative spellings were suggested.
- RxNav linked the text string to a semantic clinical drug name.
- Putative NDCs were derived.
- Images for each putative NDC were retrieved from the Micromedex drug image database (which was indexed by NDC).
- Images were displayed for user selection.

The RxNav Web services proved well suited for this function. Response time was typically 1–2 seconds, and the services were consistently available. RxNav was considerably easier to implement than downloading RxNorm tables and updating them when new versions were released. The Micromedex drug image database also generally performed well; however, it often lacked entries for putative NDCs that RxNav generated for generic medications. Images were most commonly available for solid medication forms (eg, capsules, tablets), less frequently for inhaled forms, and rarely for liquid forms.

### Dosing Frequency: Representation and Linkage to Knowledge Databases

**Figure 3:**
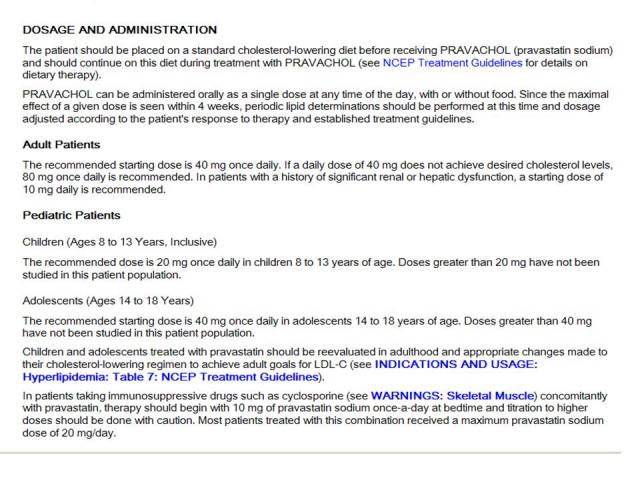
DailyMed entry for pravastatin

Representation of dosing frequency proved more challenging than representation of the medication identity. We endeavored to include representations that would facilitate machine-actionable decision support to assist users with scheduling, but resources available at the time were inadequate in several ways. One challenge was making guidelines computable. Guidelines on frequency of administration from drug knowledge databases (such as DailyMed) are available only in descriptive form, not in a codified, computable form (Figure 3). While it is reasonably easy to convert descriptions of simple frequencies into machine-actionable representations, it is much more difficult to encode important additional descriptive constraints on dosing, particularly in relation to food consumption. For example, tetracycline, a medicine commonly given to children in the MMH target user group, should be taken with a glass of water on an empty stomach, half an hour before or 2 hours after meals, and never at the same time as antacids or iron. Another challenge was capturing dosing information from prescribing data. While the National Council on Prescription Drug Programs SCRIPT standard for structured and codified SIG (dosing instructions) includes the necessary structure for basic instructions, it does not support complex instructions or timing of doses [32]. Thus, complex dosing instructions in prescribing data were embedded in noncomputable text strings.

Therefore, we needed to supplement drug knowledge databases with metadata about frequencies (Table 2) and needed to create custom rules to automate scheduling (Table 3). For the MMH prototype, primitive knowledge bases were constructed for medications commonly used in cystic fibrosis, a prototypical pediatric disease that requires complex medication regimens.

**Table 2:** Encoded dosing frequencies and metadata

Frequency	Translation	Doses per day	Spacing of doses	Comments
Q8h	Every 8 hours	3	8 hours	
TID	Three times a day (during waking hours)	3	Variable, but with doses spaced as evenly as possible during waking hours	
QHS	At bedtime	1		Some medications dosed QHS should be given in the morning for patients working a night shift
QAC	With each meal	3, but may be as needed	Variable	Dosing dependent on planned meal times

**Table 3:** Custom rules for automated scheduling

Rule	Example
“All doses of this medication have been placed on the schedule.”	A user schedules at 7:00 AM an antibiotic that is to be given every 8 hours. Doses are automatically added at 3:00 PM and 11:00 PM.
“These two doses are too close in time.”	The user above tries to move the 11:00 PM dose to 7:00 PM.
“[Medication] should be taken with every meal.”	The user is taking digestion enzymes and schedules a snack. A dose of digestion enzymes is automatically added to that time.

### Form Factors, Functions, and User Interfaces

The primary goals of the CCT, derived from the Care Transitions Intervention [17], were to assist older patients with multimorbidities by (1) helping to create medication lists using diverse prescribing and dispensing data, (2) providing easy access to authoritative consumer health information, (3) helping identify discrepancies between their personal medication list and medication lists from clinicians, and (4) preparing for visits with clinicians. A tablet PC was chosen as the primary form factor for several reasons. We sought to accommodate mobility, since medications are often stored in multiple locations in the home [33]. Touch-screen input was chosen to minimize computer anxiety [34] and to decrease input problems associated with mapping horizontal input (mouse or track pad) to vertical visualization (computer screen) [35]. We incorporated bar-code input based on acceptance of this technology by adults with complex conditions in previous work [36]. The system was implemented on a platform consisting of a tablet PC (ThinkPad X60; Lenovo, Inc, Morrisville, NC, USA) running the Windows XP tablet operating system (Microsoft Corporation, Redmond, WA, USA) with a bar-code scanner (Bluetooth Cordless Hand Scanner Series 7; Socket Mobile, Newark, CA, USA) to scan bar-codes that may be available on medication labels. The Web-accessible user interface was developed using HyperText Markup Language, PHP Hypertext Preprocessor [37], and cascading style sheets (CSS), for high performance and stylistic consistency.

The goals of MMH were to provide interconnected Web and mobile applications that would allow (1) caregivers to create a medication schedule, (2) caregivers to select medications for which use should be prompted, (3) patients to receive medication prompts on a mobile device, (4) patients to confirm that a dose was taken, and (5) caregivers to track medication-taking behavior. For the Web component, MMH was constructed to operate on any standard Web browser. It was developed using PHP with asynchronous JavaScript and XML and Flash (Adobe Systems Incorporated, San Jose, CA, USA) components for the user interface. An alert/notification system used PHP 5 and communicated with mobile phone devices using the short message service (SMS) messaging protocol.

## Results

### User Feedback during Development

While a comprehensive discussion of the iterative development process for CCT [26] and MMH [25] is beyond this discussion, a number of findings from interim user feedback sessions were particularly notable.

In general, the form factors proved to be appropriate. For CCT, older adults into their late 70s liked the concept of a mobile touch-screen tablet with large, readable text. In addition, they liked the concept of using a bar-code scanner to enter medication information from the prescription label, rather than entering the information by typing. However, the oldest users—those over 80—were averse to using any computerized interface for medication management, even when we took pains to refer to the tablet as an “appliance” rather than a computer. For MMH, children and their parents felt it was appropriate for school-aged children to carry and use a mobile device to assist in medication management. However, a proposal to embed the mobile device in a toy (such as a teddy bear) for younger school-aged children was not well received. Rather than making the device friendlier, younger children felt that carrying the toy would be stigmatizing.

Incorporating images of medications into the user interface was also greatly appreciated by both children and older adults. Both groups wanted medication images to be displayed on their respective Web interfaces. When the MMH mobile device sent medication prompts, the use of both text and medication images was greatly preferred to prompts with text alone. At the time of development, multimedia messaging service image messages were typically offered only on mobile plans at additional cost and were not integrated with SMS text messages, so the MMH prototype accommodated the desire for images by embedding URL links to images in SMS text messages.

**Figure 4:**
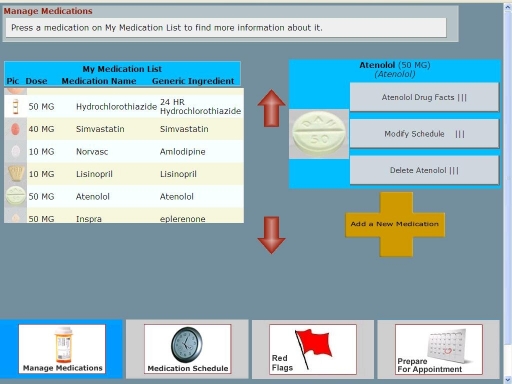
Dock navigation for Colorado Care Tablet

**Figure 5:**
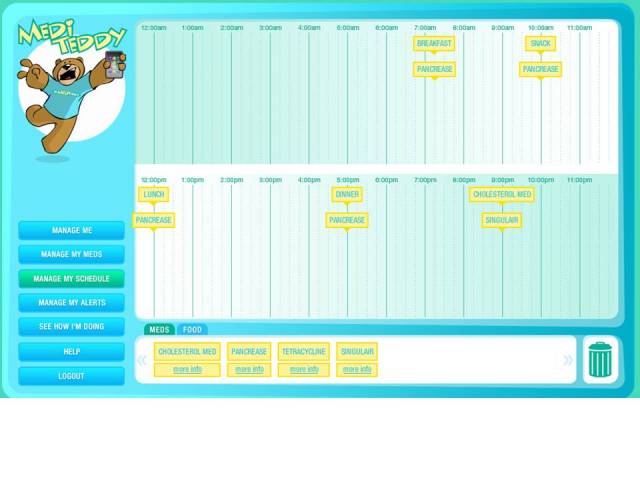
Drag-and-drop medication scheduling in MyMediHealth

Unlike younger users, older adults encountered unexpected difficulties with common user interface metaphors for navigation and actions. For navigation, older users preferred a dock of 4 key functions identified by an icon and text (Figure 4) instead of typical Web navigation structures with expanding top-horizontal and left-vertical action links. Within each core function, activities followed a linear “wizard” structure. Older adults also had problems with drag-and-drop actions when they had to schedule medications. In contrast to children and their parents, who found it very intuitive to drag medications from a personal medication list and drop them on to a calendar for scheduling in MMH (Figure 5), some older adults thought that dragging a medication from one list (for instance, one of their doctors’ medication lists) to their own list would corrupt the source (ie, would result in the medication being removed from the doctor’s list). Potential corruption of information maintained by medical professionals was a major point of concern.

As a whole, older adults consistently desired simplifications in CCT, even when this limited the application’s functionality. For instance, while it was expected that older adults would be interested in building their personal medication lists by referring to medication lists kept by their doctors, the older adults preferred *not* to be presented with multiple doctors’ medication lists. Instead, they wanted to view an aggregated list of all the medications that had been filled in the last year, from which they could select which ones were still being taken. Similarly, they found the user interface busy and confusing when their personal medication list was compared side by side with one of their doctor’s medication lists. In fact, they had little interest in ad hoc medication reconciliation at home. Instead, they felt that it was more appropriate for a medical professional to handle medication reconciliation at the time of appointments. To accommodate these preferences, the ultimate design of the CCT prototype could send a simple report of medication list discrepancies to a provider in advance of appointments.

### Application

The development process resulted in the construction of working high-fidelity prototypes for user testing (see Multimedia Appendix 1 for CCT and Multimedia Appendix 2 for MMH). CCT was evaluated by 7 users in a final videotaped task-based usability test where participants created medication lists, a list of symptoms a participant should watch for during a care transition, and a memo in preparation for a doctor’s visit. MMH was evaluated by 8 adult caregivers during a user study where participants created a medication list and developed a schedule. The MMH prototype was further subjected to evaluation by an online learning community [38]. Final testing of the high-fidelity prototypes confirmed the value of design choices made during iterative development, but also uncovered new practical issues in practice.

The ultimate design of CCT proved straightforward for users to navigate. Participants of all ages and computer skills were able to navigate CCT functions using the dock and linked wizards to build medication lists, to seek answers to common questions about individual medications, and to prepare for upcoming visits. However, users wanted CCT to answer additional questions about the medication list in general (whether there were drug interactions, whether it was dangerous to take “so many” medications, and whether some medications could be dropped). While the concept of the touch screen was well liked, many users found the touch screen insufficiently sensitive to their finger motions and required a stylus for certain tasks (such as using an on-screen keyboard). The bar-code scanner also performed inconsistently for bar-codes associated with prescription labels.

The functional MMH prototype was also well received. Overall, testers said that the scheduler prototype was generally easy to use, helpful for the family, and helpful for communication among family, school, and providers. Some members identified some important missing features, including support for dosing that varies by day or by degree of symptom, prompting about ideal locations on the schedule for a particular medication dose, support for as-needed dosing administration and dosing given less frequently than daily, and a more intuitive set of tools to create a medication list.

Other practical issues were uncovered for the mobile device. First, while the method of embedding a URL to provide both a message and a medication image was successful, it required at least 3 steps to manage an alert (receive a message alert, retrieve the message, and select the hyperlink). Second, since cell phone messages cannot be prioritized and are given bandwidth after cell phone audio calls are taken care of, there is the potential for message latency. While most messages are delivered within seconds of when the message is scheduled, some cell systems can hold a message for hours, or even not deliver the message at all. We experienced this latency intermittently during pilot testing. Although the system can be programmed to keep retrying a page until the patient acknowledges that they have either taken or not taken the medication, a page outside the correct timeframe may result in a missed dose.

We also tested integration of the two applications, using a scenario where the user used CCT to build a medication list and answer common questions about individual medications, then used MMH to set up a medication schedule. This scenario proved successful: medications entered in CCT were visible in MMH and vice versa, and it was possible to switch from one application to the other and back easily. However, due to differences in color and font, screen sizes, and user interface paradigms (touch screen vs point-and-click), further user interface development would be required to make transitions between the applications truly seamless.

Demonstrations of CCT and MMH, as well as source code for these applications (which is available under the Creative Commons license), are available from the Project HealthDesign website [39].

## Discussion

### Principal Findings

In this project, we succeeded in creating working prototypes of an interoperable PHR that accommodated fragmentation of care (using medication information from a variety of sources) and provided practical assistance in medication self-management. Employing a service-oriented architecture with shared components for data storage and information retrieval facilitated the development of complementary applications that could be tailored to different target users. Our user-centered design process allowed us to refine and simplify user interfaces to maximize usability even for relatively computer-naïve users.

### Implications of the Findings

Our development effort has implications for informatics resources supporting medication self-management applications. We found the National Library of Medicine’s online service-oriented RxNav utility for normalization of medication identities to be very useful. Since similar open-source services to provide medication images and consumer health information would also be useful, National Library of Medicine’s recent work on MedlinePlus Connect is particularly welcome. Ideally, these resources should support both prescription and common nonprescription medications. Enrichment of standards and resources related to medication regimens would also be welcome. To provide robust assistance in scheduling complex medication regimens, two areas of development are needed: (1) ongoing refinement of standards for encoding medication instructions for prescriptions, and (2) more comprehensive, codified, machine-actionable resources for dosing recommendations. Information of particular interest is shown in Table 4.

Although the working prototypes were well received, our development process also highlighted practical issues regarding appropriate form factors and user interfaces for the respective target populations. For older adults with limited computer experience, use of common metaphors (such as drag-and-drop and hyperlinked navigation) may not be appropriate. Older adults are also willing to trade off navigation flexibility and functionality if it allows for a simplified user interface. Form factors such as tablet devices and bar-code scanners can accommodate their visual and dexterity needs, but they need further refinement to be used reliably and consistently. For children, mobile phones are an appropriate vehicle for prompts and reporting, but limitations in the ability to deliver images and recognition of latency issues need to be taken into account.

**Table 4:** Desirable encoding of machine-actionable dosing recommendations

Which tablets may be crushed, which capsules may be sprinkled, and which may not Which medications may be administered by routes other than the strictly oral route How or whether to reschedule missed doses Whether a medication should be taken away from or with meals Whether two medications can be taken together

### Comparison With the Literature

Our project builds upon previous work outlining the core needs for personally entered medication data [40,41] and reports of tethered [19] and untethered [42] PHRs for medication management. It builds upon the growing literature supporting the utility of mobile phones for prompting and recording medication taking [43-47]. However, it also shows that enhancing self-entered medication lists (such as MyMedicationList [42]) with personal information from diverse sources (pharmacy aggregators and electronic health records) is far more challenging for patients than simply providing patients a view of the medication list stored in a single tethered electronic health record.

### Limitations

The primary limitation of this project is that it was not possible to provide patients with their own medication information for testing. The common PHR platform was standards-based but was designed for rapid prototyping, not secure storage. Linking the applications to secure platforms and presenting users with real medication information would allow for more realistic testing, both in the laboratory and in the field. It would also be useful to confirm our findings with larger numbers of participants in more geographically diverse settings.

### Call for Further Development

The open-source code available from the Project HealthDesign site is intended to facilitate and catalyze future development based on the concepts presented here. With the development of highly functional commercial PHR platforms such as Dossia, Google Health, and Microsoft HealthVault, each with an expanding “ecosystem” of partners sharing data, it is possible to develop functional prototypes of CCT and MMH that can be deployed in the field. MMH is being expanded to provide a suite of tools for medication management in asthma, including a patient-generated pictographic medication list, text message-based medication reminders, a printable medication administration record, and an inhaler dose counter to help ensure that refills are requested in a timely fashion. CCT could also be redeployed on the iPad, which has a clean, simple form factor and robust touch-screen interface that has been enthusiastically received by consumers. Testing its utility in the context of care transitions would be particularly valuable.

## Multimedia Appendix 1

Colorado Care Tablet walkthrough with annotated screen shots

## Multimedia Appendix 2

MyMediHealth walkthrough with annotated screen shots.

## Abbreviations

CCT: Colorado Care Tablet
MMH: MyMediHealth
NDC: National Drug Code
PHR: personal health record
SMS: short message service
