# Supplementary material for: Two Complementary Personal Medication Management Applications Developed on a Common Platform: Case Report
Source: J Med Internet Res. 2011 Jul 12;13(3):e45. doi: 10.2196/jmir.1815 (PMC3222174; doi:10.2196/jmir.1815)
Supplement: Supplementary file 1 [file jmir_v13i3e45_app1.pdf]

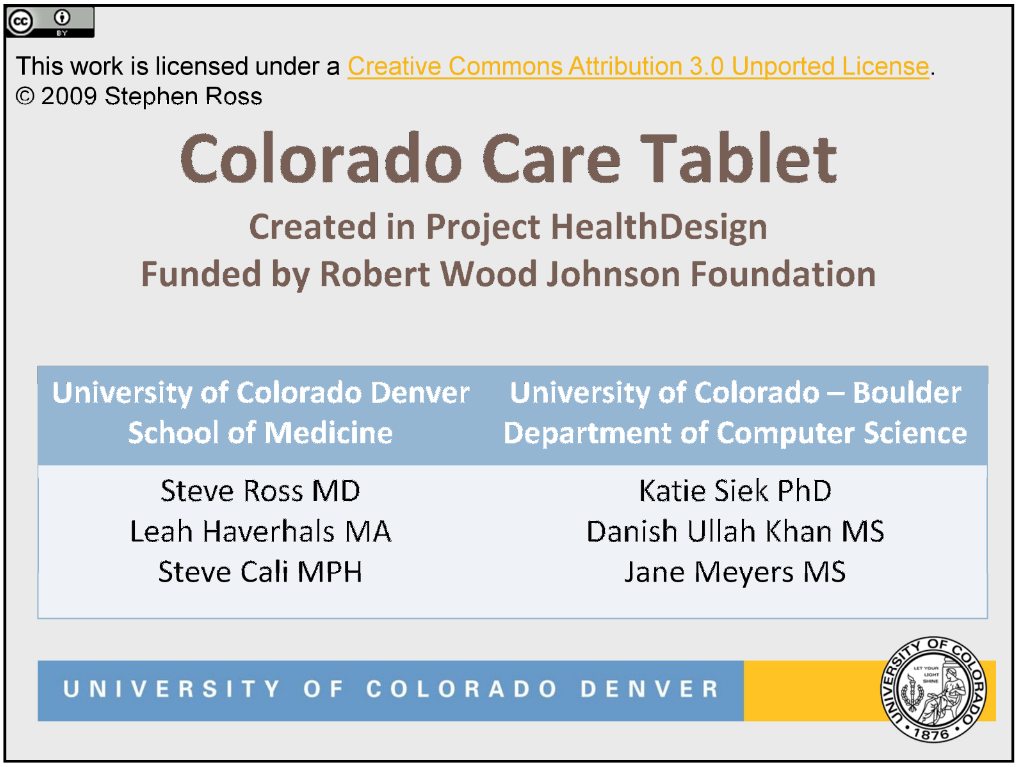

Thank you for your interest in the Colorado Care Tablet, which is a personal health record prototype developed as part of Robert Wood Johnson's Project HealthDesign. It might be more properly described as a personal health application, designed specifically for older adults with multiple medical problems who must manage transitions of care (going from one doctor to another, or being hospitalized and returning home).

The core team members for the project are listed above. Steve Ross was Principal Investigator for the project. We would also like to acknowledge the team members on our oversight group, who provided guidance on our methods and development:

- **Eric Coleman MD MPH**, UC Denver Division of Geriatrics
- **Chen-Tan Lin MD**, UC Denver Division of General Internal Medicine
- **Lisa Schilling MD**, UC Denver Division of General Internal Medicine
- **Sunny Linnebur PharmD**, UC Denver School of Pharmacy
- **Mark Ruscin PharmD**, UC Denver School of Pharmacy
- **Dana Abbey**, UC Denver Health Sciences Library

We are also grateful for the help we received from Olivier Bodenreider, Kelly Zeng, and Stuart Nelson at the National Library of Medicine that allowed us to use NLM's RxNav tools. We also appreciate Thomson Micromedex for allowing us to extend a license for drug images to be used for this project.

Finally, we would like to thank the test subjects who provided critical information throughout our user-centered design process.

This work is licensed under a Creative Commons Attribution 3.0 Unported License.
© 2009 Stephen Ross

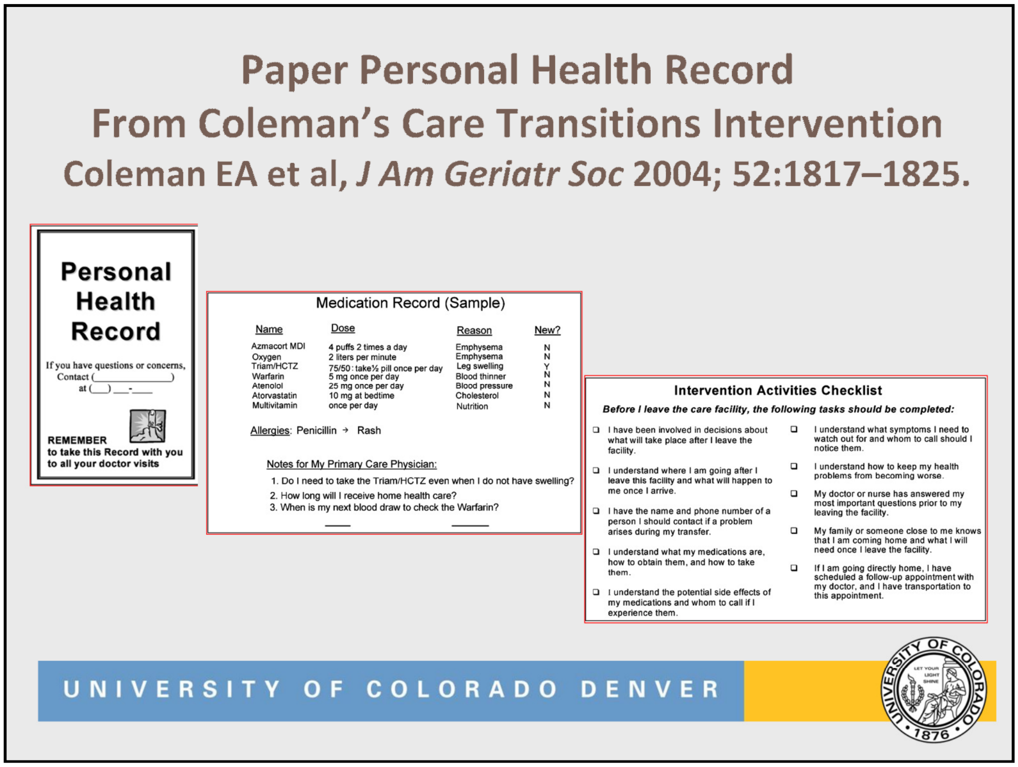

We were fortunate to have Eric Coleman MD as a Co-Investigator on our team. Dr. Coleman developed the "Care Transitions Intervention" to improve potentially dangerous transitions of care in at-risk older patients. This intervention employs a paper personal health record (parts of which are shown here) and a trained transitions coach to address four "pillars" of safe transitions:

1. Medication self-management

2. A patient-centered record

3. Primary care and specialist follow-up

4. Knowledge of ''red flags'' warning symptoms or signs indicative of a worsening condition

This intervention was shown to reduce readmissions in at-risk older persons in a randomized controlled trial (Coleman EA, Parry C, Chalmers S, Min SJ. The Care Transitions Intervention. *Arch Intern Med* 2006;166:1822-1828).

We used this highly successful program as a paradigm for our development, with the goal of creating an electronic personal health record that would be interactive and would make electronic personal health information accessible.

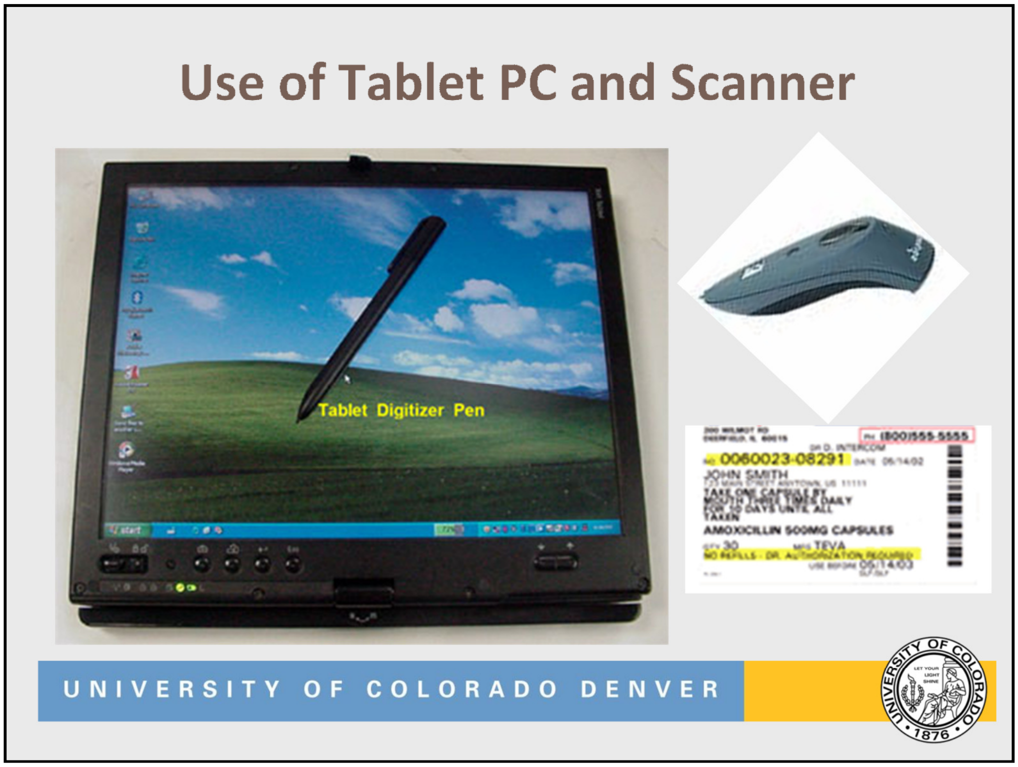

We used a tablet PC as the platform for this project. Our development platform was a Lenovo X60 tablet PC (similar to the picture) running Windows XP Tablet edition. The tablet PC was configured so that it could be operated either with a tablet digitizer pen (or stylus), as shown, or with the fingertip.

We chose this because we wanted to create a user interface that older adults with limited computer experience would find easy to read and intuitive—more of an appliance than a computer. Because our research and others' showed that people keep medicines throughout the house (e.g. morning pills by the coffee maker, evening pills at the bedside), we wanted a device that users could take where needed. But we also wanted sufficient screen space to make text easy to read for those with visual impairments, which was not possible on a smart phone. The device could stay connected while mobile by cellular web (WWAN) technology.

We also chose this paradigm because it spoke to the utility of future "smart home" or "pervasive computing" applications, in which the user interface could be projected on any surface in the home, operable by monitored hand movements.

To ease the burden of entering medication information, the Colorado Care Tablet allowed patients to enter prescription numbers from pharmacy labels (in the red box on the prescription label), rather than having to type in the name of the medication. We also included a scanner to allow alternate entry by scanning bar codes, avoiding typing altogether.

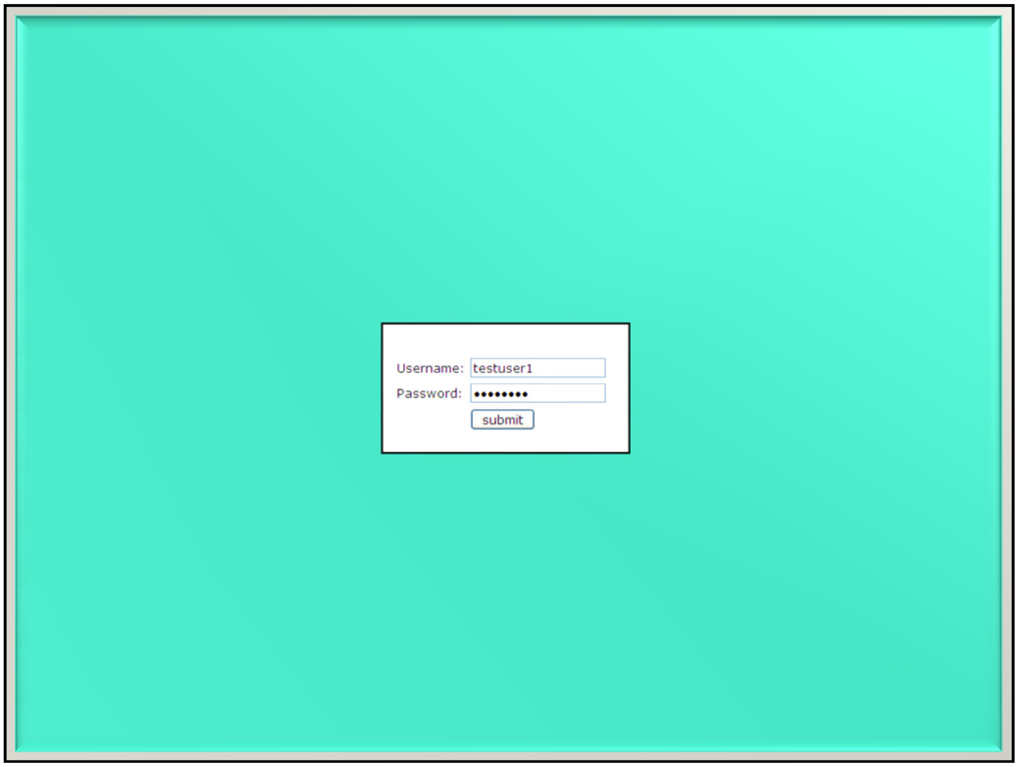

Here is the beginning of the slideshow for a use scenario for the Colorado Care Tablet.

This is the login screen. Identity management was facilitated using functions provided by the Project HealthDesign core platform, which was used for all of the Project's prototypes.

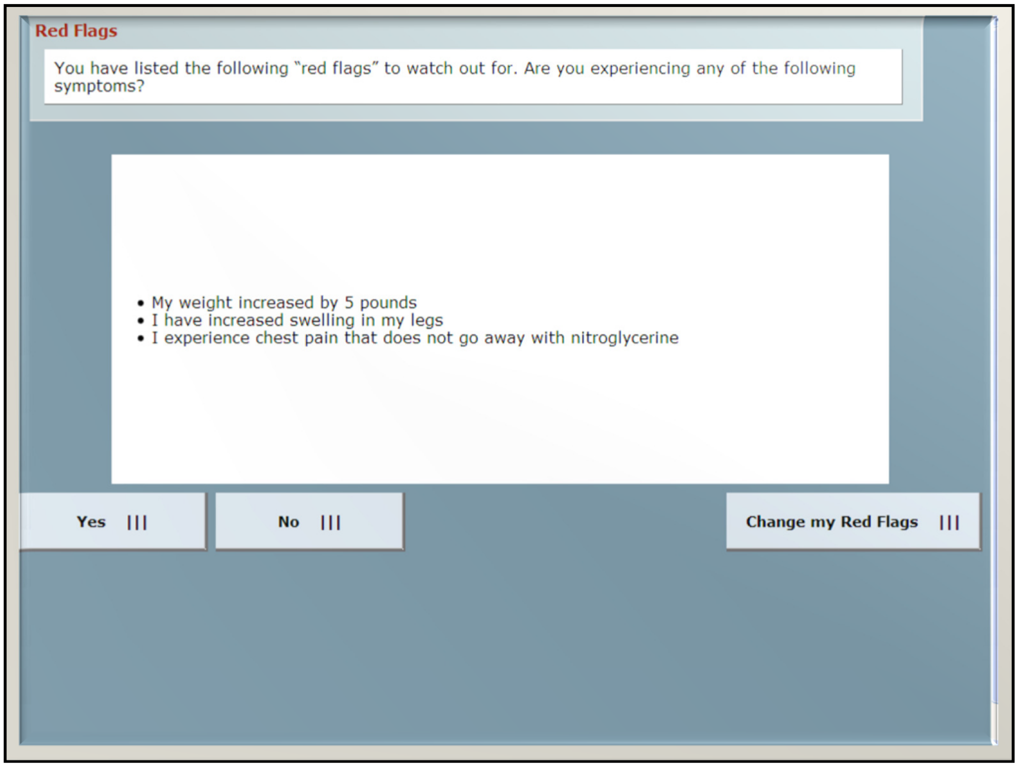

Here we present a scenario in which a user already has “red flags” (signs of decompensation to watch out for) set up. When red flags are set up, users are asked to review them right after logging in. Users who indicate that they have been experiencing these symptoms (by touching the “Yes” button) are instructed to contact one of their physicians right away.

In the future, sensors in a “smart home” might be able to automatically sense when a red flag condition (such as excessive weight gain) have occurred, and actively prompt the patient.

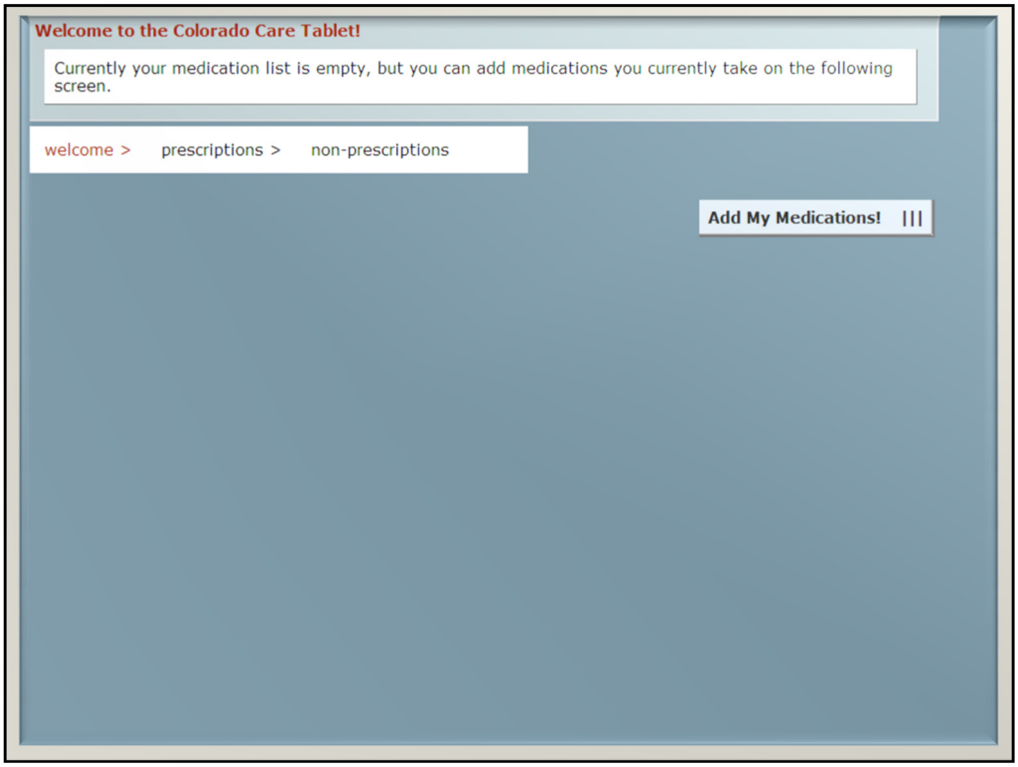

Here is the welcome page that a new user would see. We envision that an at-risk person would use the Colorado Care Tablet in the hospital before discharge, or on return home. As with the Care Transitions Intervention, it is likely that many users would be assisted by family caregivers and a care transitions coach.

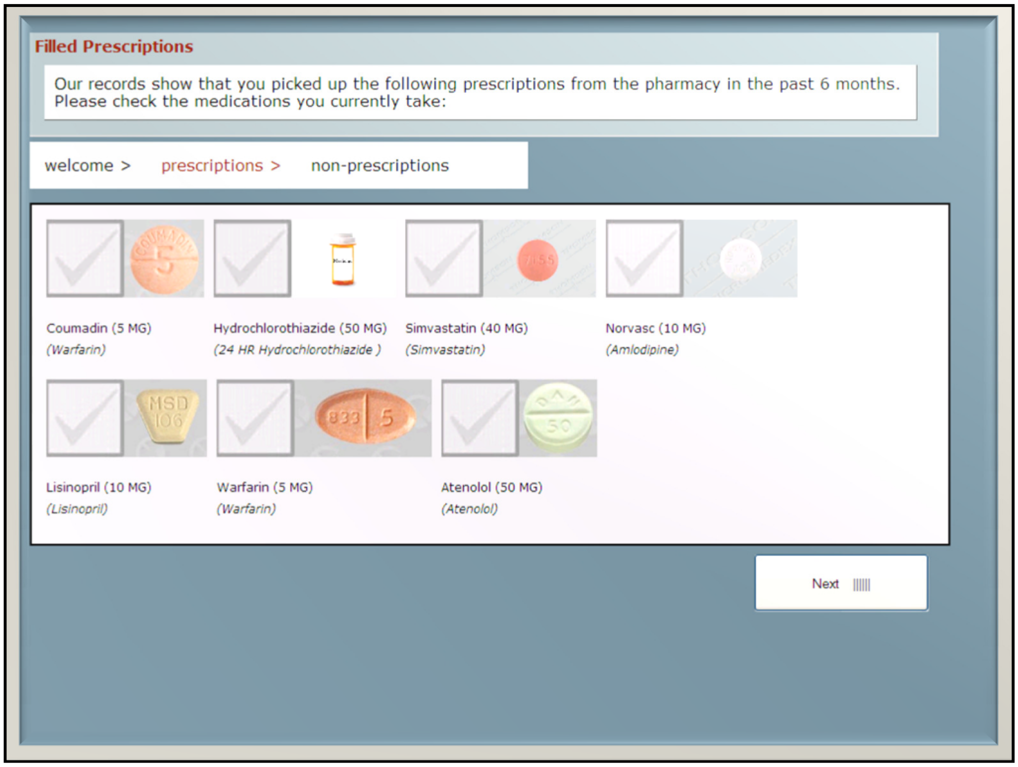

The Colorado Care Tablet walks patients through several steps to create a medication list. This screen shows the user all of the prescriptions the user has filled in the last six months. This "fulfillment" or "dispense" information is available from individual pharmacies and from aggregators like SureScripts/RxHub. (Note that information about what physicians have prescribed—available from electronic medical records—is used later in the application, as part of Prepare for Appointments function.)

The user selects medicines by pressing on the checkmark, which turns green to indicate that a selection has been made.

Note that the user is shown pictures of medications when they are available. Many users remember their prescriptions from shape and color, rather than by name. We used a database of images from Thomson Micromedex to link NDC codes (from pharmacy data) to pictures. When pictures are not available, a generic pill bottle is shown.

All medicines include both the generic name and the trade name. We employed web services (the "RxNav API") from the National Library of Medicine to link NDC codes to drug names.

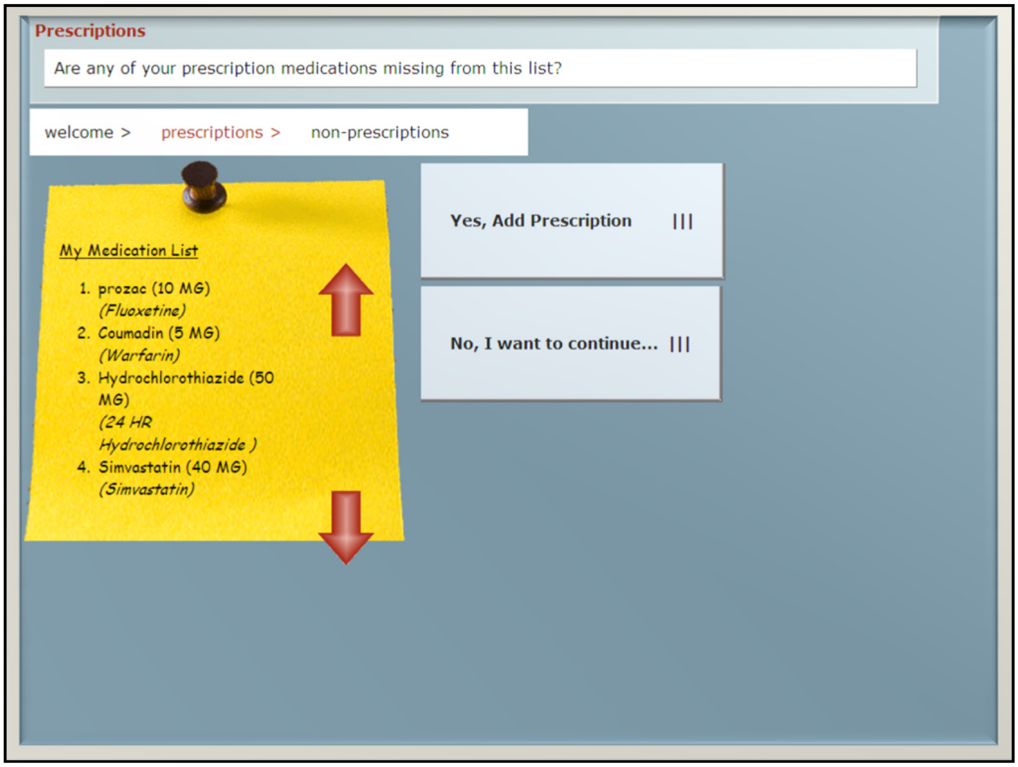

The user is now shown her medication list. The user may add additional prescriptions by touching, "Yes, Add Prescription."

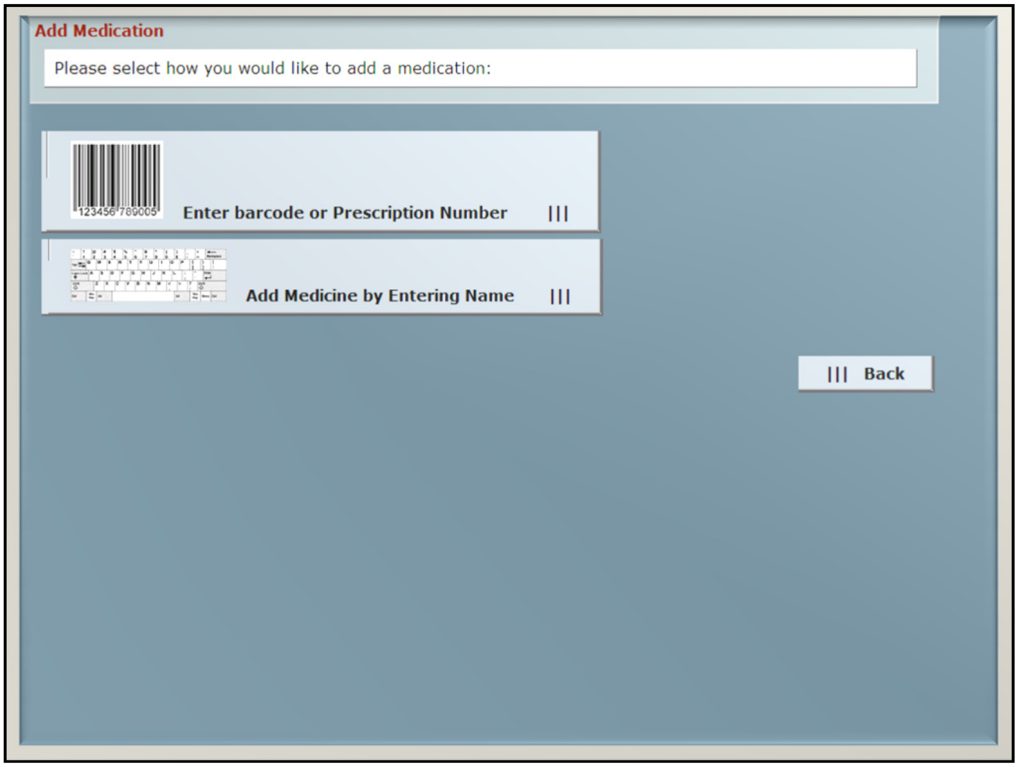

The user has two options for adding a prescription: entering a barcode (using the scanner) or typing in the name.

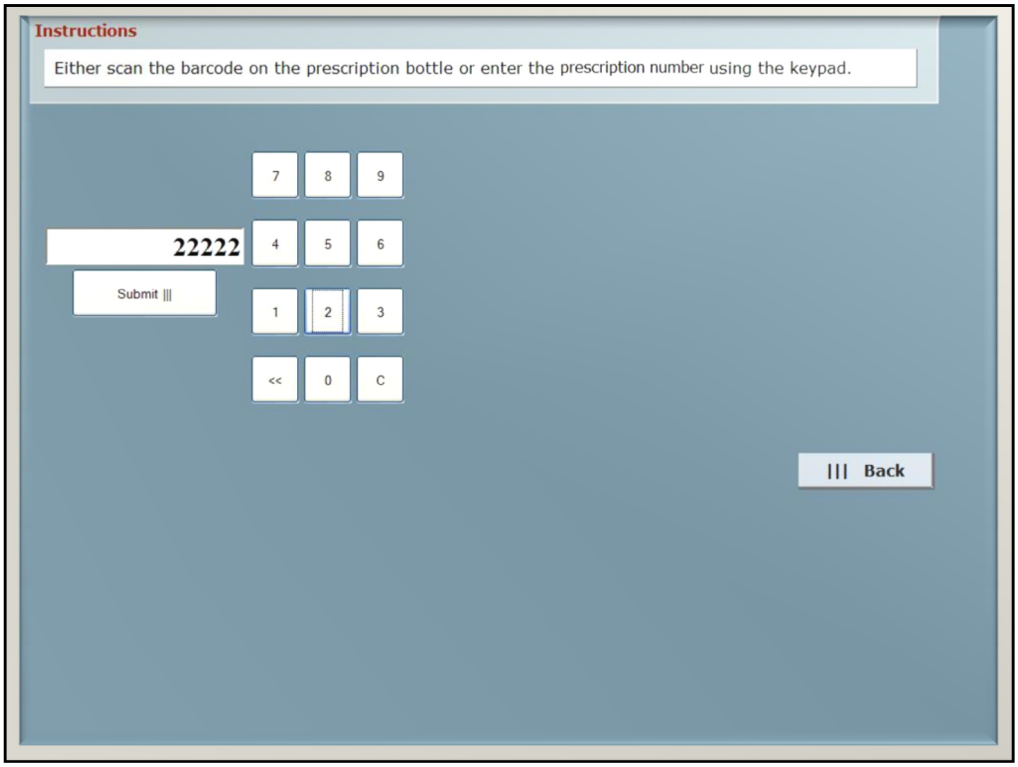

Here, the user can enter a prescription number from the medication label, or can scan the bar code from the label. The prescription number is kept in pharmacy aggregator databases like SureScripts/RxHub. Prescription numbers are also stored in bar codes.

A touch screen keypad is provided so users don't need to use a separate keyboard.

Users found the bar code scanner to be particularly easy to use to enter medications. Note, however, that many pharmacies (such as Target) do not print bar codes on their labels.

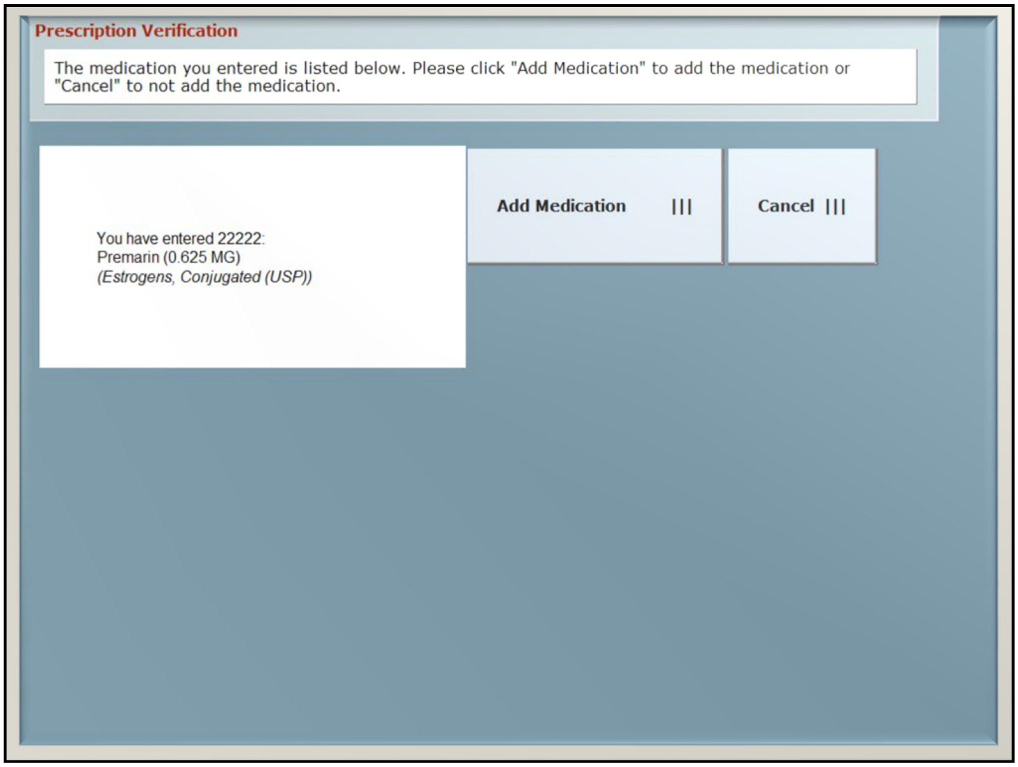

The medication matching the prescription number was found, and the user can confirm it by touching “Add Medication.”

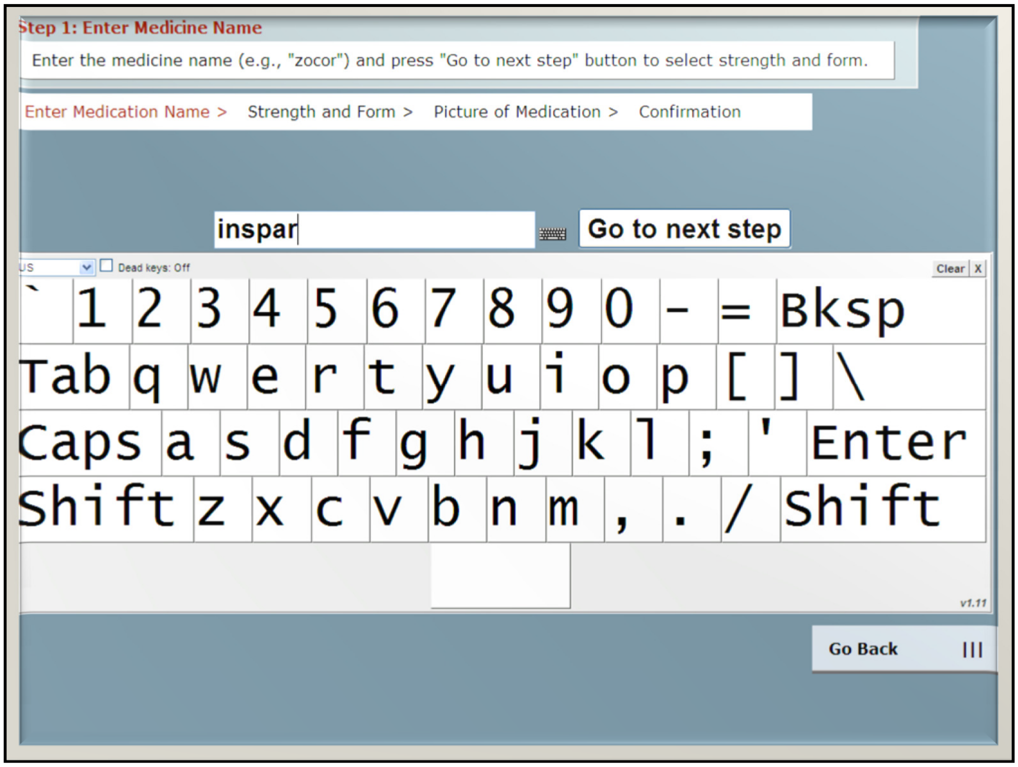

Here, the user is entering the name of a medication. Again, a touch screen keypad is presented so that a separate keyboard is not needed.

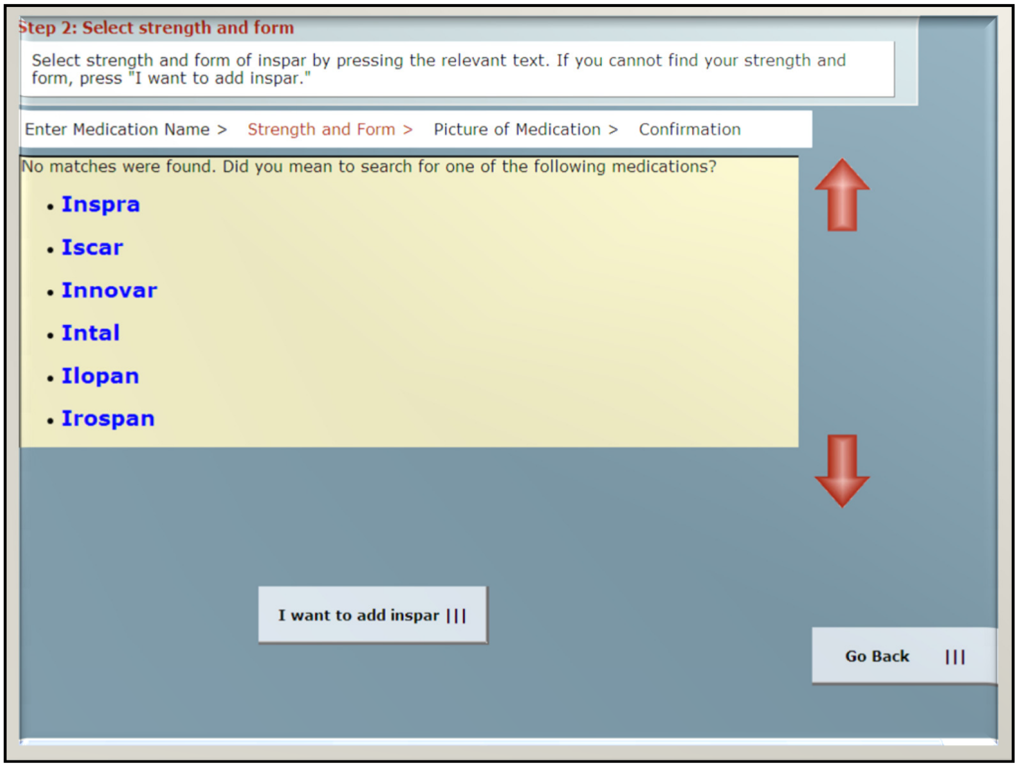

The user misspelled "Inspra" as "Inspar." Using spell check functions from NLM's RxNav APIs, The Colorado Care Tablet recognizes the misspelling, and provides alternative spellings.

The user can also stick to the original spelling. This is useful if the user wants to add medications that might not be in a pharmacy database, such as herbal remedies.

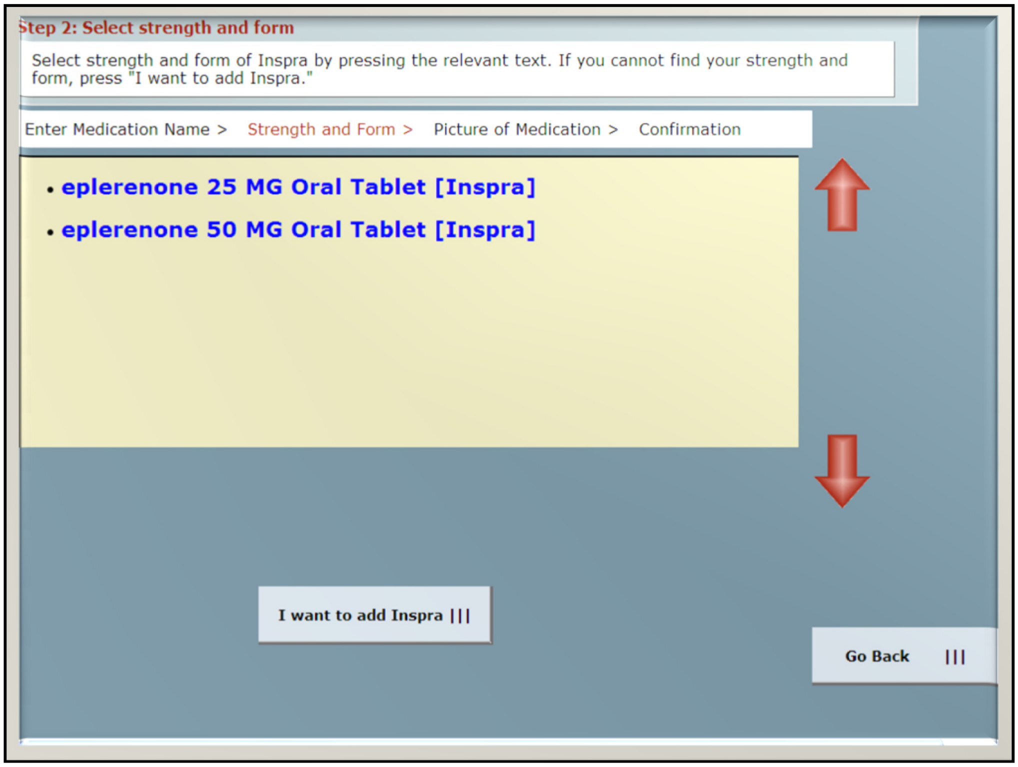

Having selected “Inspra,” the user can now select the strength and form of the medication.

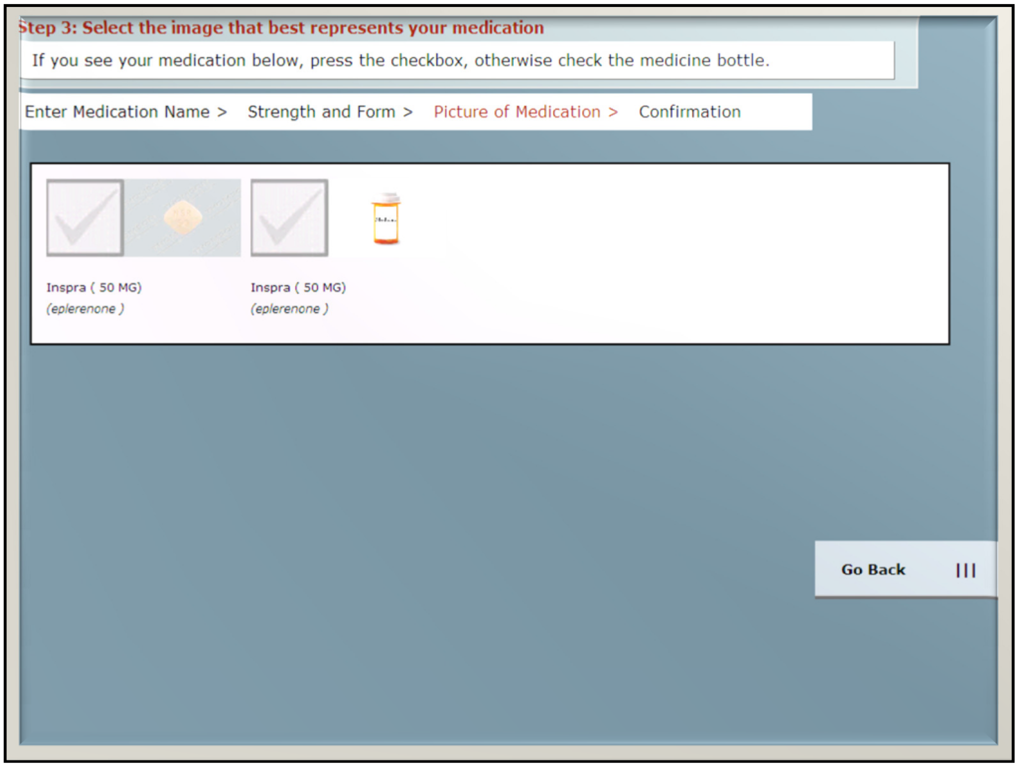

The user can now select an image of the medication. This image will stay associated with the prescription throughout the Colorado Care Tablet. This is useful when multiple images corresponding to the same name, strength, and form are available. For example, all of the following are the same active substance, but with different shapes and colors:

•Prinivil 10 mg

•Zestril 10 mg

•Lisinopril 10 mg [Mylan]

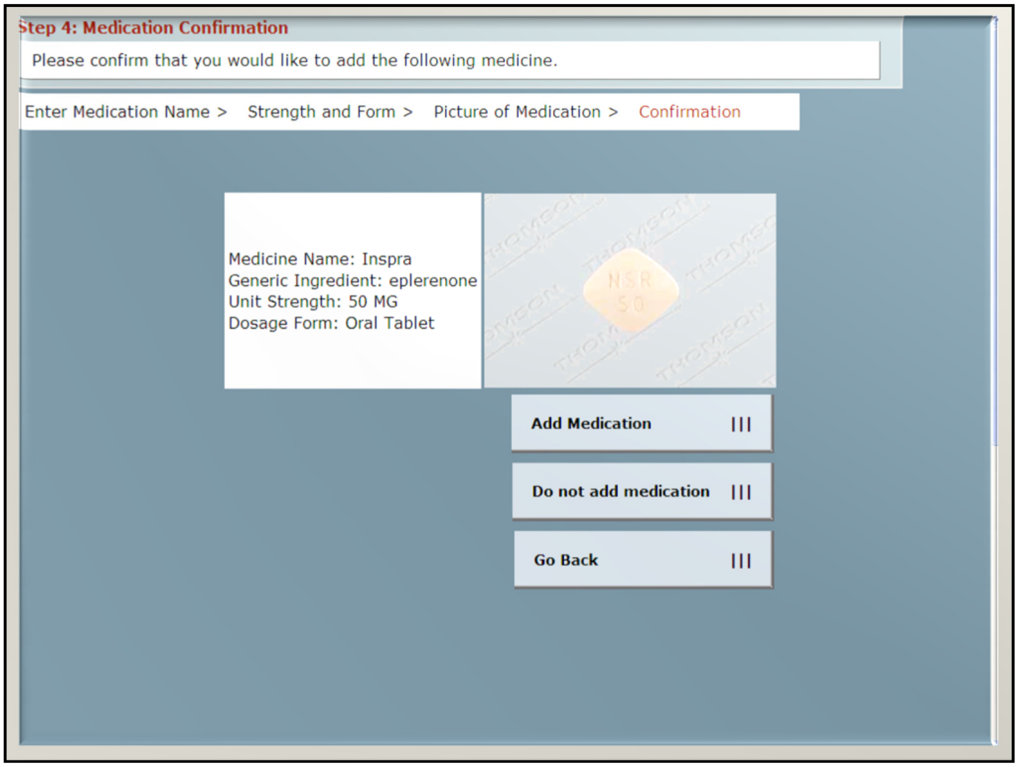

The user definitively confirms her choice to add the medication to her medication list.

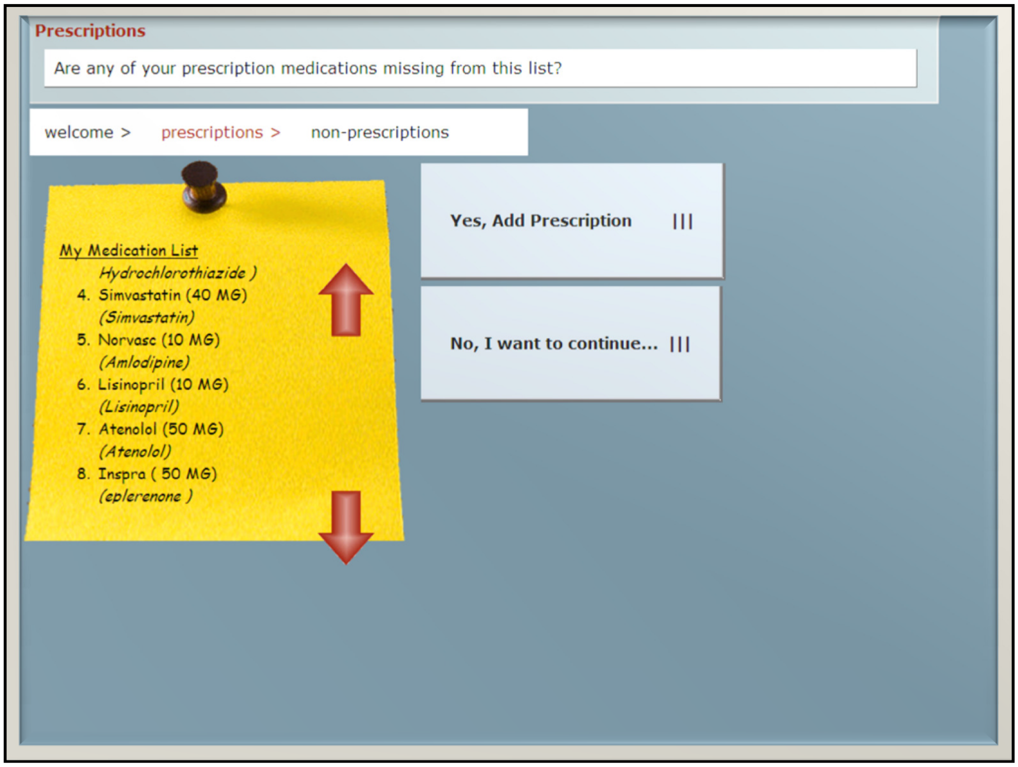

The user can continue to add prescription medications in the same manner.

Nonprescription medications can then be added, but spelling check and images may not be available for all nonprescription medications.

Once all of the medications have been added, the user touches “No, I want to continue…”

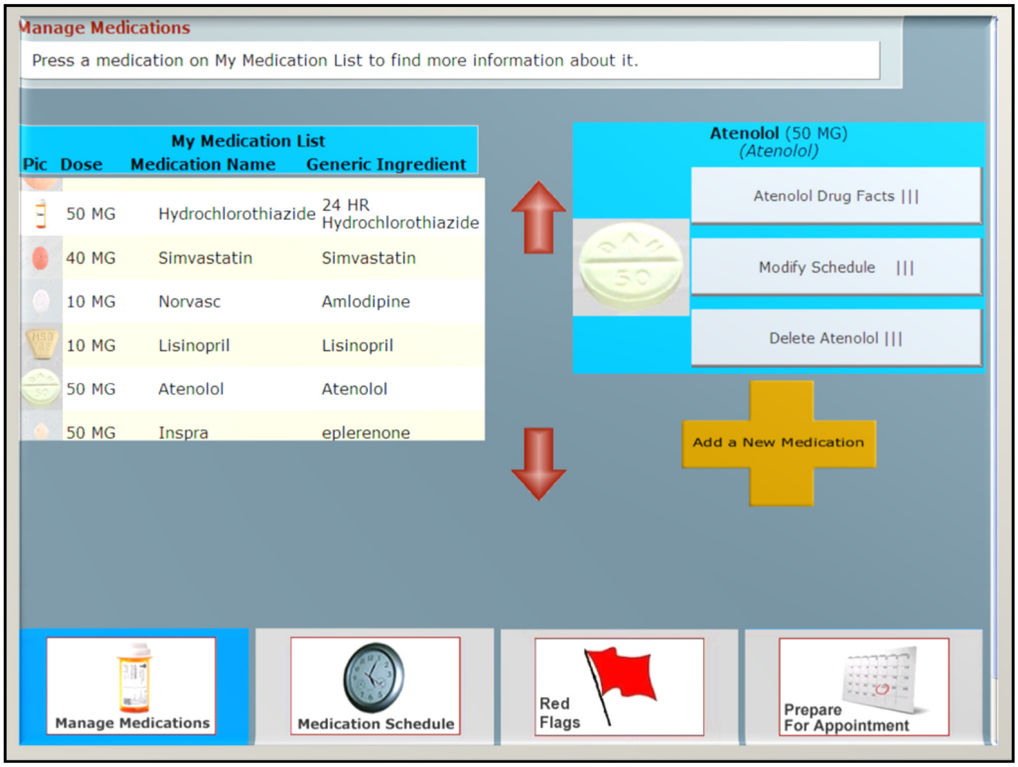

Having completed updating the medication list, the user now reaches the Medication Management page of the Colorado Care Tablet.

A dock of functions is provided at the bottom of the screen. The user can select from four functions by pressing the corresponding button on the dock. Older users unfamiliar with web browsing found this much easier to navigate than hyperlink or hierarchical schemes.

In Medication Management, the user can touch one of the medications in the mediation list. A detailed image is displayed in the upper right. The user can get more information about the medication, change how it is scheduled, or delete it.

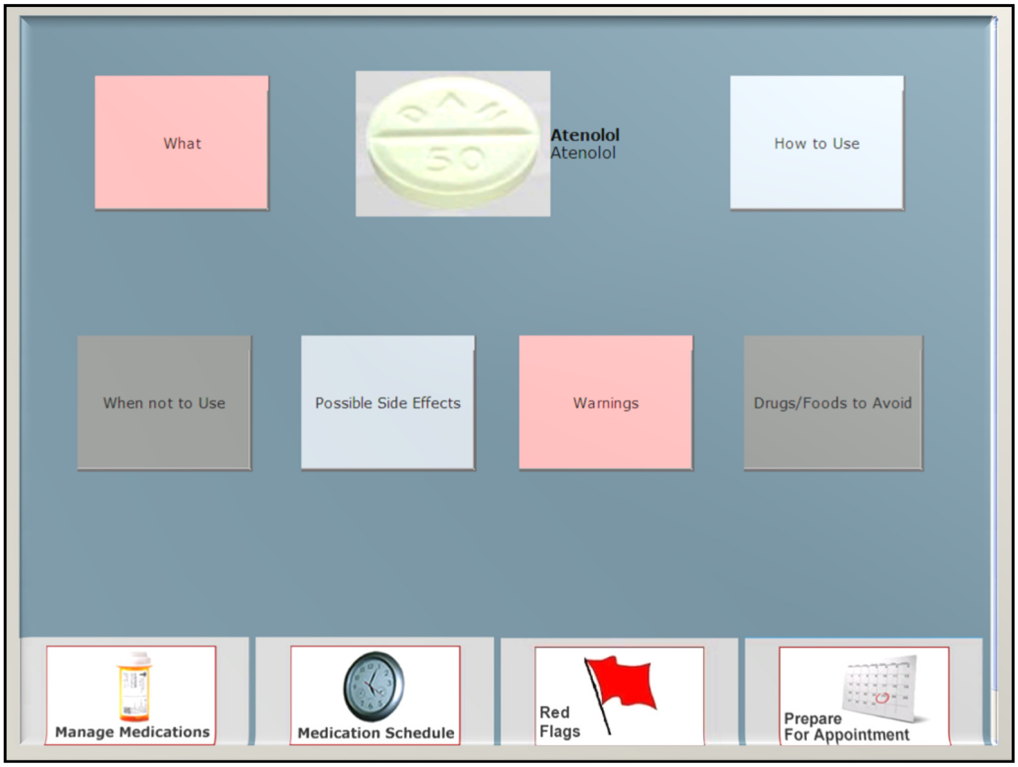

Having selected Atenolol Drug Facts, the user is now shown information about Atenolol, broken up into common topics. While younger users might find hyperlinks and scrolling for this information to be intuitive, it is useful for older adults to have the information available at the touch of a button.

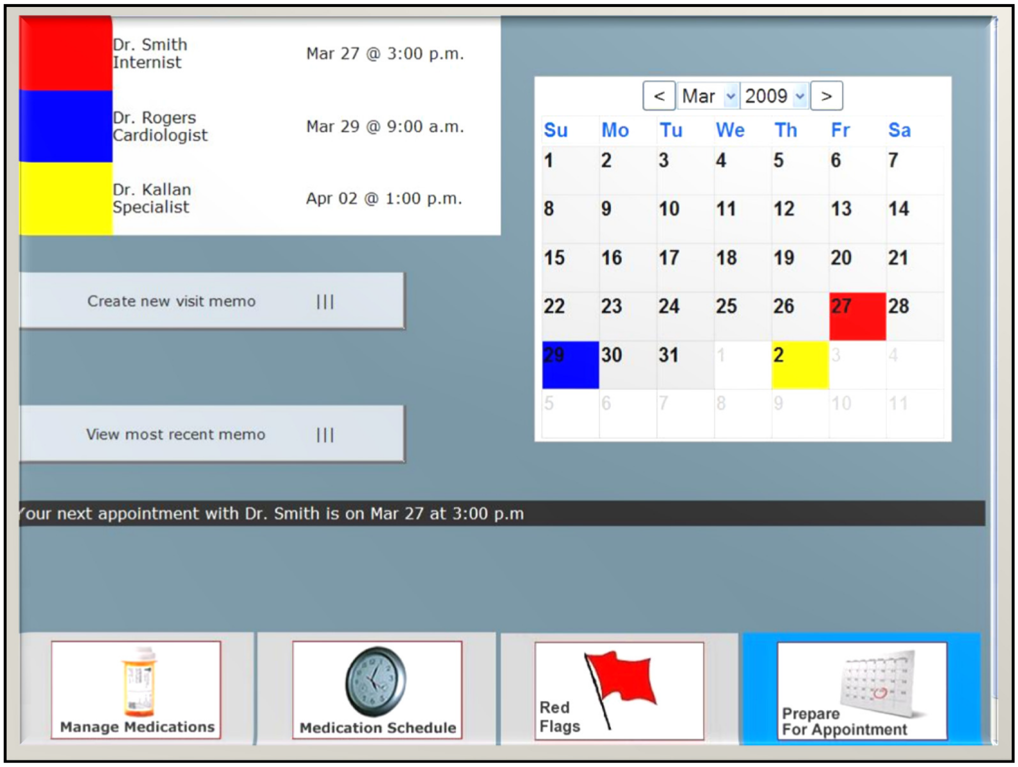

When the user selects Prepare For Appointment on the dock, she can view upcoming appointments, and can create or update a memo for that appointment.

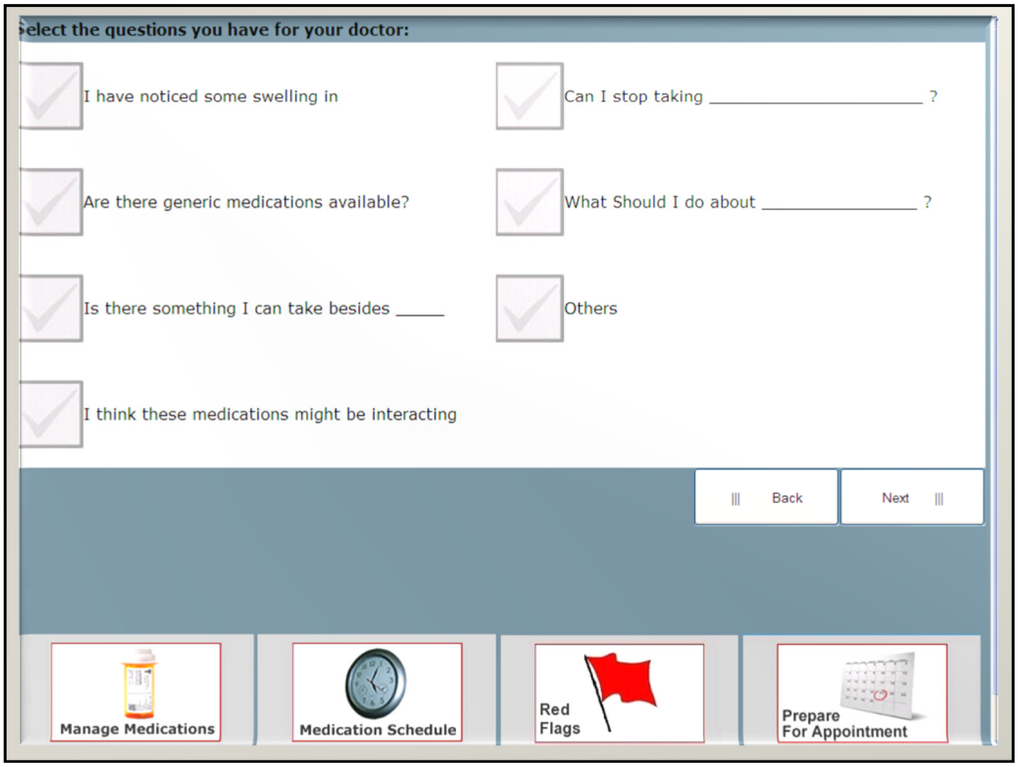

The user can select and edit common questions she might want to ask at the visit. The goal is to help patients feel comfortable asking—and remembering to ask—common but very important questions that often go unanswered at visits.

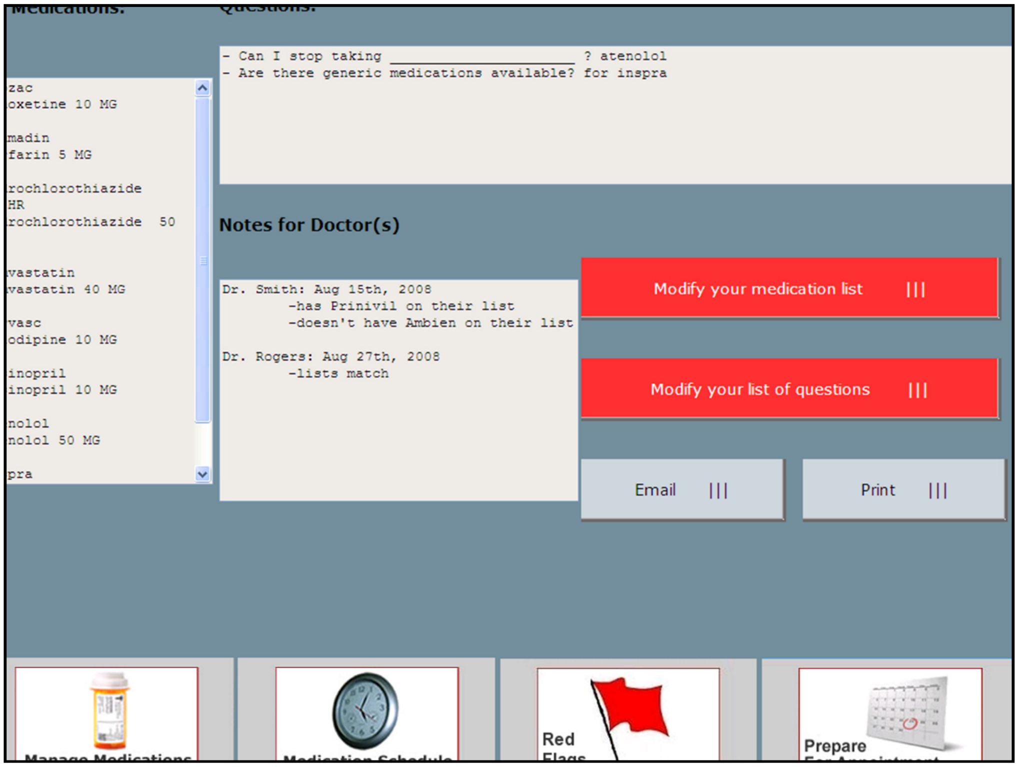

Here is the completed memo. Note that in addition to showing the questions to ask, it also shows the user's medication list, and discrepancies found between the user's list and the lists from doctors with upcoming appointments.

The memo can be printed, or sent by fax or secure electronic message to the upcoming doctors' offices. Many subjects indicated that they would also like to take the Tablet itself to their visits.

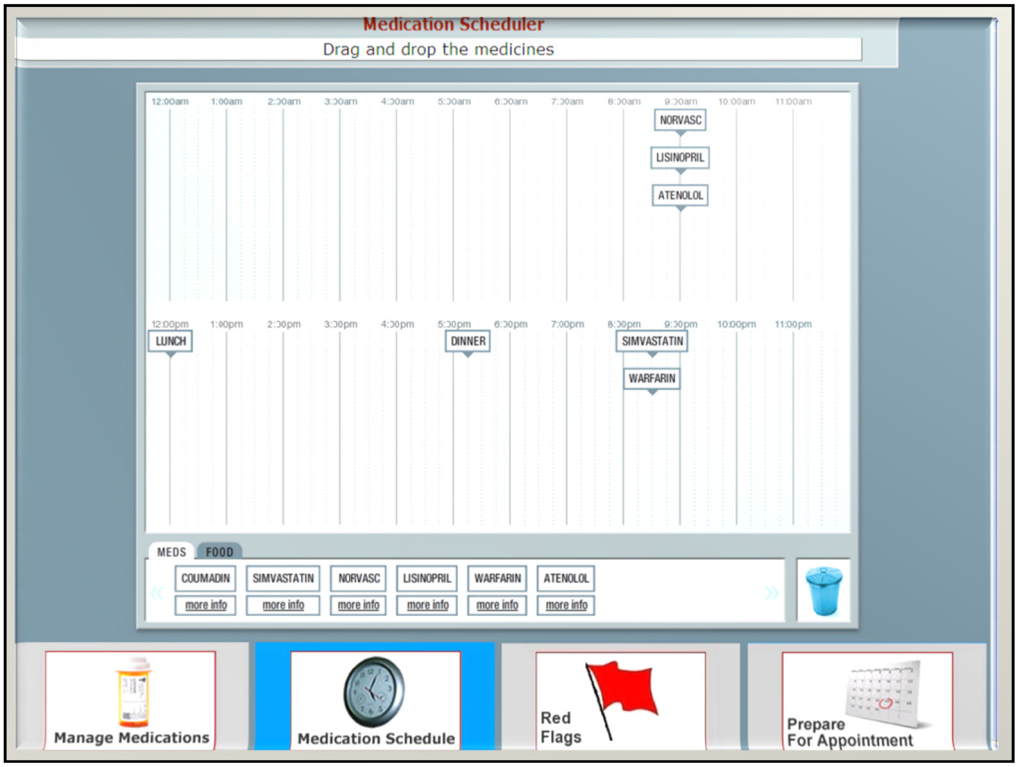

By selecting Medication Scheduler on the dock, the user can create a daily schedule of medications by dragging medications from a list on to a scheduler. The scheduler was developed by one of the other Project HealthDesign teams at Vanderbilt University Medical Center. In turn, the Vanderbilt team's application (My Medi-Health) is able to use the medication management components of the Colorado Care Tablet.

The ability for multiple personal health applications to share components and to interact with a common platform is central to the vision of Project HealthDesign.

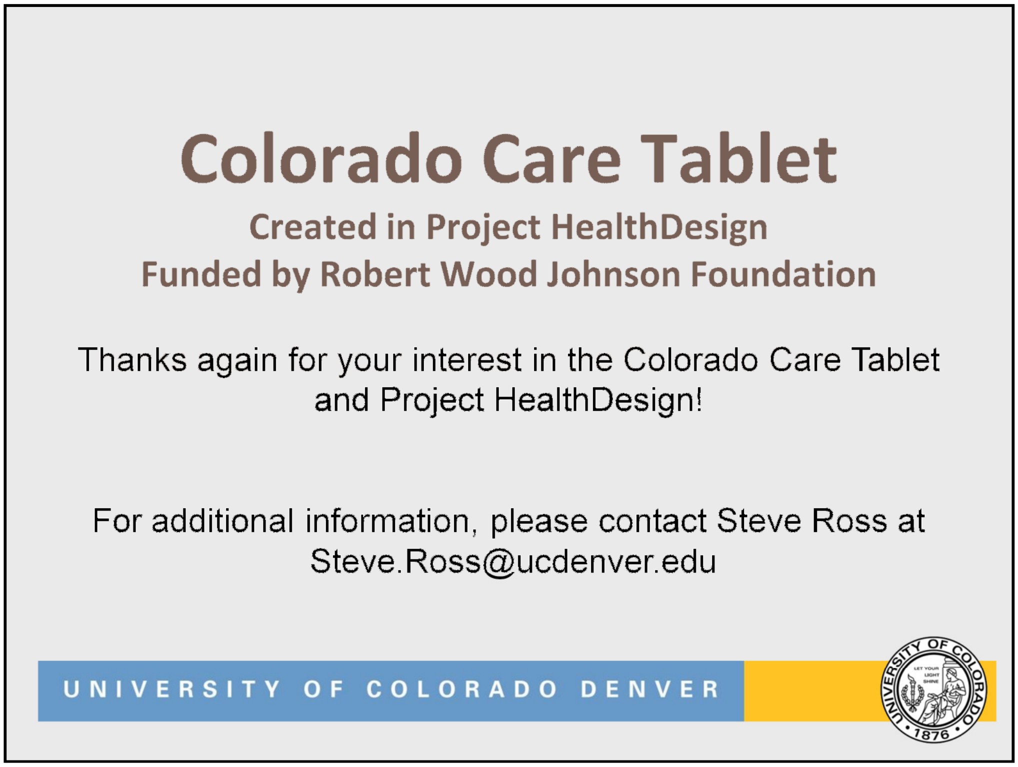

In sum, we would again like to thank the Robert Wood Johnson Foundation and the National Program Office for Project HealthDesign for this opportunity to demonstrate the power and potential of personal health records.

Using user-centered design principles to create an intuitive, accessible personal health application for older adults has been both challenging and thoroughly rewarding. Providing touch screen input with large fonts and simple navigation (with step-by-step wizards and a four-functioned dock) proved to be a winning strategy. We hope that staying close to our target users helped us minimize the dangers of simply creating an electronic replica of a paper record, or creating a personal health application that “only a programmer” or “only a medical professional” could love.

We hope that this prototype and our findings will be valuable in further development of helpful applications for this important and growing group of users.
