# Supplementary material for: Two Complementary Personal Medication Management Applications Developed on a Common Platform: Case Report
Source: J Med Internet Res. 2011 Jul 12;13(3):e45. doi: 10.2196/jmir.1815 (PMC3222174; doi:10.2196/jmir.1815)
Supplement: Supplementary file 2 [file jmir_v13i3e45_app2.pdf]

MyMediHealth System Overview

Navagation Screeen

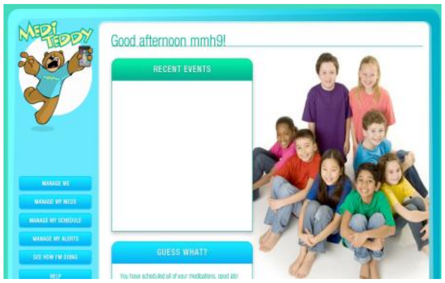

This is the opening screen for MyMediHealth. From this screen, patients use the buttons to the left to manage their profile, their medication list, their medication schedule, and the medication administration results.

MMH My Information screen

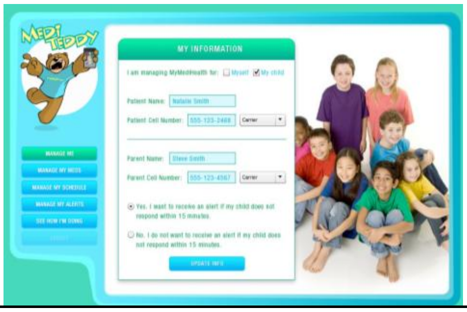

This is the information screen, where patients are able to enter their information, or if being managed by a parent, their child’s information.

Schedule Screen

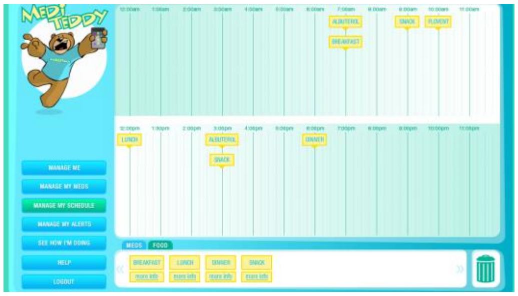

When the user selects the Manage My Schedule, he or she is taken to this screen. Medications from the patient’s medication list are listed at the bottom, and may be dragged and dropped on the schedule at a location corresponding to the time of administration. If the medication is written to be given with meals, it is automatically attached to meals. If there is more than one dose prescribed daily

MyMediHealth System Overview

Medication Screen

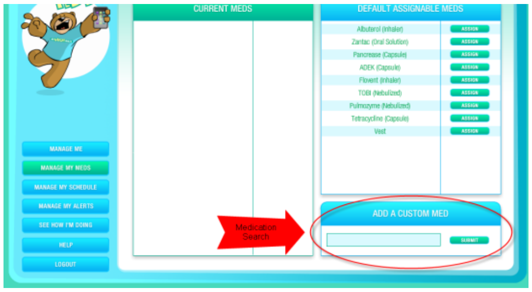

From this screen, patients are able to add medications to their medication list. They may either select a commonly prescribed medication (left) or enter a name of a medication (bottom, arrow).

Medication Search Screen

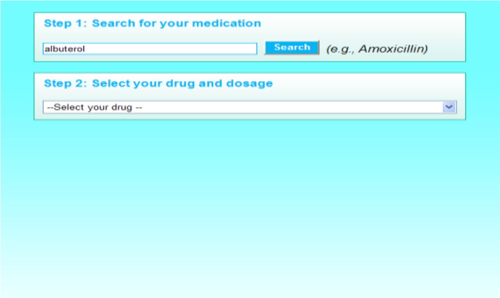

Once a medication is entered, the patient is taken to this screen to identify the formulation and an image of the medication they are taking.

Medication Screen Including Current List

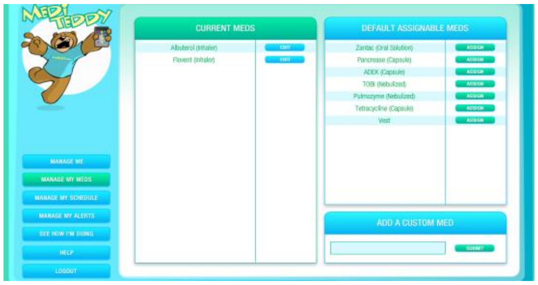

They are then returned to the medication list.

MyMediHealth System Overview

How To Turn on Alerts

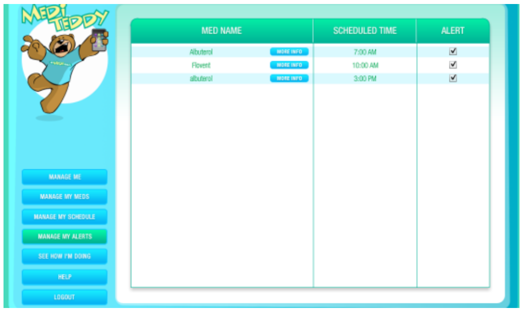

Each medication dose that is scheduled is associated with an alert. However, the user is able to turn off individual dose alerts.

Medication Tracking

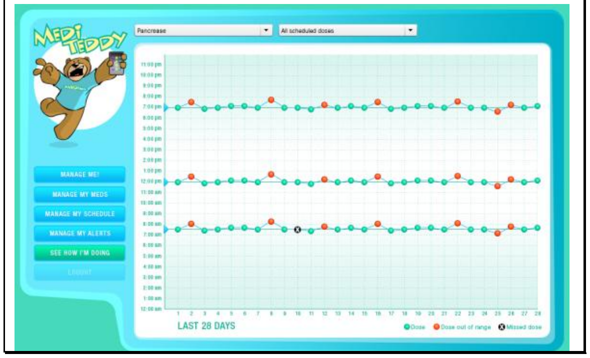

MyMediHealth provides the results of alerts (via a bi-directional interface with the phone's text messaging features) as a medication administration record.

Tracking a Specific Medication

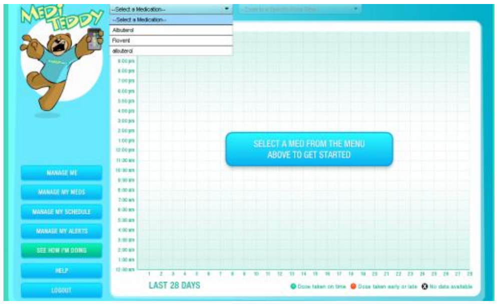

This medication administration record may be viewed for each medication on the medication list.
